# Retraction Note: Enhanced resistive switching memory characteristics and mechanism using a Ti nanolayer at the W/TaOx interface

**DOI:** 10.1186/1556-276X-8-419

**Published:** 2013-10-22

**Authors:** Amit Prakash, Siddheswar Maikap, Hsien-Chin Chiu, Ta-Chang Tien, Chao-Sung Lai

## Retraction

This article is retracted.

The journal editors would like to apologise for the early publication of the original article [1], which is being retracted as it was published prior to the completion of essential revisions.

The revised version of this article has now been published, and is available online [[2]].
